# A SOA-Based Platform to Support Clinical Data Sharing

**DOI:** 10.1155/2017/2190679

**Published:** 2017-05-25

**Authors:** R. Gazzarata, B. Giannini, M. Giacomini

**Affiliations:** ^1^Department of Informatics, Bioengineering, Robotics and System Engineering (DIBRIS), University of Genoa, Via Opera Pia 13, 16145 Genoa, Italy; ^2^Healthropy s.r.l., Corso Italia 15/6, 17100 Savona, Italy

## Abstract

The eSource Data Interchange Group, part of the Clinical Data Interchange Standards Consortium, proposed five scenarios to guide stakeholders in the development of solutions for the capture of eSource data. The fifth scenario was subdivided into four tiers to adapt the functionality of electronic health records to support clinical research. In order to develop a system belonging to the “Interoperable” Tier, the authors decided to adopt the service-oriented architecture paradigm to support technical interoperability, Health Level Seven Version 3 messages combined with LOINC (Logical Observation Identifiers Names and Codes) vocabulary to ensure semantic interoperability, and Healthcare Services Specification Project standards to provide process interoperability. The developed architecture enhances the integration between patient-care practice and medical research, allowing clinical data sharing between two hospital information systems and four clinical data management systems/clinical registries. The core is formed by a set of standardized cloud services connected through standardized interfaces, involving client applications. The system was approved by a medical staff, since it reduces the workload for the management of clinical trials. Although this architecture can realize the “Interoperable” Tier, the current solution actually covers the “Connected” Tier, due to local hospital policy restrictions.

## 1. Introduction

In the last few decades, clinical trials (CTs) have covered most of the research activities in medical fields, since they are indispensable tools for evidence-based medicine [1]. The workflow of a CT, which can be considered a particular type of a medical research project, is formed by a concatenation of several steps: planning, design, clinical data management (CDM) (data collection, data processing, and data analysis), presentation, interpretation, and publication [2]. This complex sequence requires the participation and collaboration of a multidisciplinary team [3]. Among these professional figures, the clinical data manager, who is responsible for data collection, cleaning, and management, represents a critical role [4], particularly in the case of multicentre CTs [5]. The clinical data manager must ensure that data are collected, validated, and completed consistently according to the study protocols, but also followed the indications provided by the Clinical Data Interchange Standards Consortium (CDISC) [6]. To facilitate the use of electronic technology in the context of existing regulations for the collection and interchange of data source in clinical trials, the CDISC founded the eSource Data Interchange Group (eSDI) [7]. This group leveraged standards to facilitate regulatory compliance for the acquisition, exchange, and archive of electronic clinical trial data, through the formulation of twelve requirements and the corresponding recommendations. These prerequisites are the following:

1. “an instrument used to capture source data shall ensure that the data are captured as specified within the protocol,”
2. “source data shall be accurate, legible, contemporaneous, original, attributable, complete and consistent,”
3. “an audit trail shall be maintained as part of the source documents for the original creation and subsequent modification of all source data,”
4. “the storage of source documents shall provide for their ready retrieval,”
5. “the investigator shall maintain the original source document or a certified copy,”
6. “source data shall only be modified with the knowledge or approval of the investigator,”
7. “source documents and data shall be protected from destruction,”
8. “the source document shall allow for accurate copies to be made,”
9. “source documents shall be protected against unauthorized access,”
10. “the sponsor shall not have exclusive control of a source document,”
11. “the location of source documents and the associated source data shall be clearly identified at all points within the capture process,”
12. “when source data are copied, the process used shall ensure that the copy is an exact copy preserving all of the data and metadata of the original” [7].

The eSDI group proposed five technology-independent scenarios to guide stakeholders in developing new ICT (information and communications technology) solutions for capturing eSource data able to meet the reported requirements. These possible situations are the following:

- “the storage of eSource at the investigative site,”
- “use of an eSource system provider (contracted supplier),”
- “the Single Source Concept (leveraging standards to enter eSource data simultaneously into an electronic health record (EHR) system or system at a site and a clinical study system, electronic data capture (EDC) or database),”
- “eSource extraction and investigator verification (using electronic health records),”
- “direct extraction of clinical trial data from the EHR, as an alternative to acknowledge the ultimate vision for research-healthcare data flow.”

In 2006, the eSDI stated that these scenarios were forward-thinking and that they could lead to the reuse of EHR content in future CTs, enhancing data sharing between healthcare systems and clinical research [7]. Prior to 2006, there was little use of ICT in clinical research. The few examples of computerization systems were not able to support a comprehensive clinical research platform. This was due to many obstacles in the integration of patient care and clinical research data [8]. In those years, the most common way to conduct a computer-based CT was the “capture” of clinical data in eSource documents (e.g., “ad hoc” spreadsheet files), stored at both the sponsor and investigator sites, in an attempt to emulate the paper case report form (CRF). This situation represented the first scenario indicated by the eSDI group [9]. Next, generation solutions (second scenario) proposed the replacement of these documents with dedicated interfaces, developed by a “trusted third party,” to store clinical content in trial-specific databases for research purposes only [10]. Examples of these special purpose registers were developed at national and international levels to collect specific clinical information on particular diseases [11–13]. The use of these “ad hoc” solutions reflected on the subsequent increase in the physicians' workload, due to participation in multiple clinical trials [10]. Moreover, the increased human involvement during data entry caused relevant input errors that could significantly influence the results of the studies [14].

To solve these problems, interoperability, between EHR and EDC systems, was introduced from the third scenario proposed by the eSDI group; in this vision, one-time data entry was employed to simultaneously feed both the EHR and EDC systems. In this way, data were only entered once and could then be used for multiple purposes, such as patient care and clinical research [15, 16]. This kind of approach reduces the time required for data entry and reduces user error input, since physicians do not need to copy data multiple times in different systems. Different examples of successful implementations of the single source concept are described in the literature. A generic solution was proposed by Kovalchuk et al. [17], formed by different connected components: a centralized clinical data management system (CDMS) to support clinical research, a central resource registry that managed the information collected from pervasive devices (Central Resource Management System—CRMS), and healthcare information systems, such as the EHR. Another similar solution was the one proposed by Lu [18], which is a basis for the XIENCE global post marketing study, carried out in the United States and India. In both countries, agents shared secondary data through web technologies adopting a system supported in nearly 300 sites. That system also provided some basic reporting functionalities and query management features for both the sponsor and site users.

Two other examples were recently set up by the authors at a regional level to provide infectious disease and by ophthalmologist physicians of different hospitals with one-time data entry CDMSs, developed according to their specific requirements. They both consisted of one central database and a corresponding web interface [19–23]. El Fadly et al. proposed an architecture for the integration of clinical research data entry with patient care data entry, applied in the cardiovascular radiology ward [24]. A CDMS for cardiovascular radiology was designed to provide a single data capture form, avoiding error-prone data entry in both the EHR and CDMS, while saving time. This solution presented several limitations, since it was only possible to ensure syntactic interoperability between two “ad hoc” applications, developed specifically for two existing software packages, used in that hospital ward. Another similar solution was proposed by Kush et al. [25], where the same limitations were presented. These projects demonstrated that patient care data can be successfully reused for research scopes, but this kind of approach had all the restrictions described above. Moreover, simultaneous population of EHR and EDC systems does not allow retrieval of past clinical data for new CTs (as it does not provide any tool for data search in the past). For this reason, the eSDI group introduced the extraction and investigator verification approach (fourth scenario), which is based on direct communication between the EHR and CDMS. In order to effectively achieve interoperability between clinical care and clinical research domains, it proposed bridging the HL7 (Health Level Seven) Version 3 (v3) Clinical Document Architecture (CDA) Release 2 (R2) [26], standard for EHR, and the CDISC Operational Data Model (ODM) [27], standard for CTs. In 2004, CDISC and HL7 began a collaboration with the National Cancer Institute and the FDA (Food and Drug Administration) to develop a domain analysis model called BRIDG (Biomedical Research Integrated Domain Group) [28]. This model contains representations of clinical research data with underlying mappings to the HL7 Version 3 RIM (Reference Information Model). An example of semantic integration between clinical care and clinical research contexts has been done within an existing clinical trial called ARCADIA. This study focused on treatment modalities for hypertension caused by fibromuscular dysplasia, carried out at the Georges Pompidou European Hospital (HEGP) [29]. In this context, some more general EU and US initiatives were promoted, aimed at improving the direct connection between clinical practice and research. Examples of these projects are the European Infrastructure for Translational Medicine (EATRIS) [30] and Informatics for Integrating Biology and the Bedside (i2b2) [31], which were both intended to identify the best approach to adopt these standards in order to facilitate the connection.

The eSDI group stated that when clinical data stored within the EHR were extracted for research purposes, the overall validation must be guaranteed; the investigator should check the extracted data to prove that they accurately corresponded to the EHR content (e.g., through electronic signature). The effective direct EHR content extraction (without manual validation step) was only introduced as a fifth scenario to indicate a possible future solution that would be used if additional regulations could facilitate this process [7]. In 2008, the Direct Data Extraction approach was subdivided into four different tiers with corresponding requirements by the EHR4CR (Electronic Health Records for Clinical Research) Functional Profile Working Group. The aim was “to expand and adapt the functionality of EHR and associated systems, networks, and processes to support clinical research” [32]. Tier 0, called “Core” (minimum requirements), was the basic level, which involved the electronic extraction of data from the EHR and their transfer to the research system. In “Connected” (Tier 1), the use of a standard was considered to enable automatic extraction. The “Integrated” level (Tier 2) extended the previous layer with a fluent stream of data flowing in both directions, from EHR to the research system and vice versa. The last and most futuristic level was “Interoperable” (Tier 3), which showed a complete ensemble of EHR and the research system, as they were located on the same international network, allowing data sharing. Starting from these requirements, in 2009, the EHR4CR Functional Profile Working Group proposed addressing some regulatory considerations specific for this field to adopt the EHR system as an eSource for CTs; in particular, specified deidentified data could be extracted for clinical research [33]. Nowadays, although EU (European Union) legislation does not yet provide for a common regulation for the reuse and sharing of health data for research purposes, 17 of 28 member states adopted specific national laws on this topic; the other 11 countries only have general data protection legislations [34]. In an Italian context, the importance of this topic was further acknowledged with a dedicated law, currently in operation, which attested that one of the aims of the establishment of patient health records (PHR) was for medical, biomedical, and epidemiologic studies and research [35, 36].

This paper presents a standardized SOA- (service-oriented architecture-) based solution that the authors designed to realize Tier 3, the “Interoperable” layer, defined by the EHR4CR Functional Profile Working Group. This architecture surpasses the concept of direct extraction of clinical trial data from the EHR, which represents the fifth scenario indicated by the eSDI, supporting effective clinical data sharing between the hospital information system (HIS) of the care facilities and registers or CDMS of research centres, both involved in CTs.

## 2. Background

The IEEE (Institute of Electrical and Electronic Engineers) defined interoperability as the ability of two or more systems or components to exchange information and to use the information which has been exchanged [37]. Interoperability can be divided into three levels: technical, semantic, and process interoperability [38, 39]. All three aspects of interoperability are interfering: semantic interoperability requires technical interoperability while process interoperability requires semantic interoperability [40].

### 2.1. Technical Interoperability

Technical interoperability is the ability to move data from one system (A) to another (B). It defines the degree to which the information can be successfully “transported” between systems [40]. The service-oriented architecture (SOA) represents the design strategy most adopted to support technical interoperability between multichannel and composite real-time applications implemented within large-scale distributed environments [41]. The main reason for the diffusion of the SOA paradigm is that it proposes a highly feasible approach to promote the easy integration and alignment of new and existing solutions into a cohesive architecture, all with minimal impacts to service consumers with a resulting highly reduced economic cost [41–44]; for these reasons, this approach was successfully adopted in distributed healthcare architectures [45]. A SOA is formed by a set of network-accessible and platform neutral software services (web services), which can encapsulate the functionality and information of existing applications and provides them through well-defined interfaces [41–43]. This aspect makes the SOA suitable for the healthcare scenario where the reuse of software, which has been financed by previous investments, is a fundamental element to be approved by healthcare organizations [46, 47]. In detail, a web service is a networked system, which is able to interact by using standard application-to-application Web protocol (Simple Object Access Protocol (SOAP)) over well-defined interfaces. These interfaces are described through a standard functional description language (Web Service Description Language (WSDL)) to represent an abstract model of what the web service offers to client applications. Through a WSDL document, the abstract description shares the data types used by the application through XSD (XML Schema Definition) files and defines both the messages and the application interfaces, which represent collections of operations exchanging those messages [48, 49].

### 2.2. Semantic Interoperability

The semantic interoperability ensures that both systems understand the data in the same way: the information sent is unaltered in its meaning [40]. Unlike technical interoperability, which is realized with common technologies in all IT sectors, semantic interoperability depends on the specific application field. In health informatics, this interoperability level between the EHR and the systems that produce and process the structured clinical data stored within the EHR is guaranteed by the adoption of standards to manage both syntax and semantics, produced by different international initiatives. The standardization efforts in syntax include HL7 v3 CDA R2 [26], European Committee for Standardization (CEN)/International Organization for Standardization (ISO) 13606 [50], and openEHR [51, 52]. The choice of which standard to use mainly depends on the recommendations provided in each single country [53]. For example, in 2010, the Italian Healthcare Ministry produced national guidelines for the Italian institutional EHR and recommended the adoption of HL7 v3 CDA R2 [54]. In all cases, for the management of clinical content semantics, standardized vocabulary such as LOINC (Logical Observation Identifiers Names and Codes) [55], SNOMED (Systematized Nomenclature of Medicine) [56], and ICD (International Classification of Diseases) [57] must be adopted.

The CDA R2 provides a standardized structure to create clinical documents for interchange proposes. A CDA R2 document is an XML file, which can therefore be wrapped within the SOAP message body, formed by two parts: a header and a body. The header contains contextual information (the patient, the author, the custodian, the authenticator, the type, etc.) while the body presents the clinical report, which can either be enclosed within a NonStructuredBody or within a StructuredBody. A NonStructuredBody is a simple container for any random file (PDF, HTML, Word, jpeg, etc.) where the information content is not semantically represented. To allow the whole CDA content to be effectively computer processable, a StructuredBody must be used. It includes an arbitrary number of sections, and, in the case of complex documents, more sections can be components of other sections. In turn, each section has a “narrative block,” which represents the content expressed using human language within specific XML tags, and a variable number of entries, which codify the content using the mentioned standardized medical vocabularies and HL7 v3 data types. In particular, each entry nests can have one or more ClinicalStatements, which can be one of the following: an Observation, a RegionOfInterest, an ObservationMedia, a SubstanceAdministration, a Supply, a Procedure, an Encounter, or an Act. If necessary, a ClinicalStatement can be related to another one through a semantic relationship (e.g., reason, cause, and component) or be referred to an ObservationRange, only in the case of Observation, or an ExternalActChoice, which can be one of the following: ExternalAct, ExternalObservation, ExternalProcedure, or ExternalDocument [58].

This structure is extremely generic and flexible and is therefore adaptable to satisfy the requirements of different interoperability scenarios. For this reason, an Implementation Guide (IG), which constrains the CDA R2 specification, must be provided for each used case. The CDA R2 Implementation Guide (CDA R2 IG) should include different chapters such as (a) scope and requirements, (b) textual and processable expression of constraints, (c) references to standards and templates used, (d) CDA R2 full instance and/or fragment examples, (e) external and internal vocabularies used/allowed, (f) use of registries, and (g) extensions. The IG is usually produced by HL7 International, then each country-specific HL7 affiliate organization is authorized to edit a national version appropriate for the local healthcare context. The choice of IG is related to the clinical and administrative data that are used for the CTs, which in turn depends on the particular class of patients considered by each implemented solution.

### 2.3. Process Interoperability

Lastly, process interoperability enables business processes and organizational housing systems A and B to work together. It defines the degree to which the integrity of workflow processes can be maintained between systems. This includes maintaining/conveying information such as user roles between systems [40]. The process interoperability requirement is satisfied when a process is compliant with standards which allow it to reach its own objective, irrespective of the propriety, location, version, and design of the IT systems used [59]. To address this need in e-health, the Healthcare Services Specification Project (HSSP) was promoted [60]. The HSSP was formed in 2005, by HL7 International and the Object Management Group (OMG), in order to define health industry SOA standards that promote effective interoperability between applications and distributed and heterogeneous devices, which belong to independent sociohealth system organizations. It is important to highlight that the HSSP is not intended to replace existing systems or implementations, but to create interface standards for a service-oriented layer to expose those healthcare assets and resources within an organization that are needed to meet business or medical needs. In detail, the aim of every HSSP project is standardization of the interface of a specific service, which is related to a functional sociohealth domain, as a generic service. All HSSP standards are distributed through the HL7 Service Functional Model (SFM), which provides a service interface specification at a functional level, and the OMG Service Technical Model (STM), which specifies the technical requirements of the service [61, 62]. In details, each OMG STM is formed by a set of human readable specification documents (i.e., pdf) and some computer processable files (i.e., WSDL and XSD) that can be used to automatically generate the interfaces adopted by web services and client applications to interact.

Two examples of products derived by the HSSP effort are represented by the Retrieve, Locate, and Update Services (RLUS) Release 1 (HL7 SFM and OMG STM are, resp., available at [63, 64]) and the Identification and Cross-Reference Service (IXS) Release 1 (HL7 SFM and OMG STM are, resp., available at [65, 66]) standards. The RLUS standard provides the description of a web service interface, called “RLUS Management and Query Interface,” through which information systems belonging to different healthcare organizations can access and manage clinical information mapped with a specific semantic signifier. The HSSP defines a semantic signifier as “the manifestation of a computable information model, tagged with a name and version and capable of being used and enforced by reference.” The RLUS Management and Query Interface is described in a specific WSDL file [64] and provides a set of functions (Get(), List(), Locate(), Put(), Discard(), and Describe()) schematically represented in Table 1. Capabilities written in bold and underlined are needed by the reader to understand the content of the following sections. A Windows Communication Foundation (WCF) service compliant with RLUS specifications was designed and developed by some of the authors within a previous European project (CHIRON) to manage clinical data interchange in a cardiac telemonitoring environment [42]. The CHIRON (Cyclic and person-centric Health management: Integrated appRoach for hOme, mobile and clinical eNvironments) project aimed to propose an integrated framework for person-centric health management throughout the complete care cycle. The mentioned WCF represented the middleware to integrate the data collected in the patients' home and the hospital environment. Another secondary aim of the CHIRON project was to facilitate the coexistence of the EHR standards (CDA R2, European Committee for Standardization (CEN)/ISO 13606, and openEHR) in a real application environment. The authors focused their activity on this target [42].

The IXS standard aims to uniquely identify and index various kinds of entities (patients, providers, organizations, systems, and devices) both within and across health organizations. It details, it defines two web service interfaces, called “IXS Management and Query Interface” and “IXS Admin Editor Interface,” described by specific WSDL files [66], through which healthcare applications and enterprises can search, create, retrieve, merge, and manage entity data whose traits are mapped with specific semantic signifiers. The IXS Management and Query Interface provides the following operations: RegisterEntityWithIdentity(), CreateIdentityFromEntity(), UpdateEntityTraitValues(), RemoveIdentity(), GetEntityTraitValues(), FindIdentitiesByTraits(), ListLinkedIdentities(), and ListUnlinkedIdentities(). The functions exposed by the IXS Admin Editor Interface are LinkEntities(), UnlinkEntities(), MergeEntities(), UnMergeEntities(), ActivateEntity(), and DeactivateEntity().  Table 2 schematically presents all functionalities provided by the IXS Management and Query Interface and the IXS Admin Editor Interface, reporting a nontechnical description (summarized description column) as well as the technical aspects (aim, input parameters, and output parameters column).

## 3. Materials and Methods

In order to design and develop an “Interoperable” Tier system, the authors decided to combine the solutions described in the Background able to completely support interoperability. The SOA paradigm was adopted as a design strategy to sustain technical interoperability because it allows the planning of large-scale distributed architecture where new and existing solutions can be integrated and aligned [41–44]. From the implementation point of view, the author used the Windows Communication Foundation (WCF) framework [67].

To support semantic interoperability at the syntax level, the authors decided to use the CDA R2 standard as indicated by the Italian Healthcare Ministry, but it is important to highlight that the proposed solution can also support the exchange of information mapped using other standards (e.g., openEHR archetypes), as discussed in the following sections. In this study, clinical trials on patients affected by infectious diseases (e.g., human immunodeficiency virus (HIV) or hepatitis B/C virus (HBV/HCV) and degenerative eye diseases were managed by a standardized SOA. In the first case, the clinical information included specific blood tests, while in the second case, the managed information was related to data collected during specific medical visits (such as the status of the vision and the objective description of the retina situation). In both cases, administrative data were represented by year of birth, gender, nationality, race, and patient identifiers. Since IG for encounters in an Italian context had not yet been produced by HL7 Italy, the authors decided to start the implementation of a solution to manage the first class of patients mentioned above. After this, the HL7 Italy IG for Laboratory Reports [68], which represents the Italian localization of the IHE (Integrating the Healthcare Enterprise) Laboratory Technical Framework [69], was used to constrain the StructuredBody content. According to this guide, a laboratory report is mapped within *laboratory specialty sections* and identified by its LOINC [55] specialty code. Each *specialty section* can contain either a single *laboratory data processing entry* or a set of *laboratory report item sections* (including in turn one or more *data processing entries*). The first case is used to map a single test (e.g., glucose concentration in serum or plasma) while the second is chosen to manage a battery of tests (e.g., complete blood counts). In both cases, the *data processing entries* contain the observations of the specific value recorded in a machine-readable format. To manage semantics, the LOINC vocabulary was adopted to translate laboratory terms.

Finally, the HSSP indication was considered for the design of the proposed architecture presented in the Results in order to guarantee process interoperability; in detail, IXS and RLUS standards were used. To support the clinical data workflow of this specific use case, the only entity which had to be managed was the anonymized patient. For this reason, the authors decided to design a Patient Identity Service (PIS), compliant with the IXS, which used the Patient class of the CDA R2 as the semantic signifier. In order to store the administrative data, the authors designed and developed a database hosted on Microsoft SQL Azure. The structure of this database was based on the Patient class of the CDA R2, which was used to map the identities, and the relations (link and merge) between the identities. As mentioned in the Background, some of the authors designed and developed a WCF service, compliant with RLUS specifications, within a previous project to manage clinical data interchange in a cardiac telemonitoring environment [42]. The authors decided to adopt the same design idea and approach to plan a Health Record Management Service (HRMS) to support the communication between the actors, which produce data, the HIS of care facilities, and those which process information, external clinical registers, or CDMSs of research centres. For the HRMS, the adopted semantic signifier was the CDA R2.

The HRMS projected for this research work differs from the one proposed previously [42]. The WCF service adopted in the telemonitoring project was located on a server (Single Processor Quad Core Xeon 3470, 2.93 GHz, 6 GB Random Access Memory (RAM), and 64 bit Windows Server 2008 R2 Standard Edition), and performance tests showed that it completely fulfilled the requirements indicated by medical staff for the telecare scenario at a prototypal level [42]. In order to adopt the HRMS within a real distributed medical application, the authors decided to host all services on a private cloud. Windows Azure is a Microsoft cloud platform, which provides high availability, scalability, and manageability, fault tolerance, geo-replication, limitless storage, and security on the cloud. It is able to detect hardware failures and to automatically move application codes to a new machine in order to allow applications to remain available to clients [70]. It allows the adoption of load balancing to implement failover that is the continuation of a service after the failure of one or more of its components. All components are monitored continually, and when one becomes nonresponsive, the load balancer is informed and no longer sends traffic to it. Load balancing also enables other important features such as scalability [71].

From an architectural point of view, the authors' previous research work was formed by a HRMS which was only connected to passive agents, that is client application [42]. This type of architecture could support data reuse with the duplication of information, but it was not sufficient to realize the “Interoperable” level. In fact, in order to allow data sharing among the involved actors, all systems must provide access to the information, which is produced and stored in specific repositories. For this reason, the authors decided to integrate new types of agents, that is, web services, as passive actors that provide access to the repository content. Furthermore, for the design of these interfaces, the authors adopted IXS and RLUS standards. In this new architectural approach, the HRMS and PIS collaborated to orchestrate the data sharing, working as intermediaries for other RLUS and IXS web services. In detail, the authors decided that HRMS and PIS would play the role of full mediators and that the web services would only communicate with the HRMS and the PIS that would compose and collect data coming from the RLUS and IXS web services. In addition, the authors decided to eliminate the database, directly connected with the HRMS, which temporarily stored CDA R2 documents. In fact, the temporal storage performed by the HRMS was useless in supporting the “Interoperable” level. The only information that the HRMS needed to know to orchestrate were the hospitals and registers/CDMSs involved and the endpoint of the corresponding RLUS and IXS web services. For this reason, the authors designed and developed a specific database hosted on Microsoft SQL Azure to store these data.

In their previous research work [42], some of the authors planned and developed client applications that were ex novo implemented within the project. In the proposed solution, the authors had to integrate new and existing systems, an aspect that represented the most critical part of the work. The existing systems involved were the HISs and the registers/CDMSs. From the HIS side, the authors considered the components which stored clinical and administrative data: the EHR and PAS (Patient Administration System), respectively. The first implementation of the solution proposed in this manuscript was focused on the management of HIV patients, and the clinical information were related to laboratory reports, as mentioned above, which were stored within the Laboratory Information System (LIS). From the research centre side, a CDMS or register was formed by a repository, which collected all data from previous CTs.

The access to the information stored in all these repositories was afforded by HIS and register/CDMS administrators with its own strategy that differed for several technicalities, but two main categories could be singled out: (1) repository access through a nonstandardized interface formed by one or more RESTfull (Representational Transfer State) or SOAP web services and (2) direct access to the database. In the first case, the communication between the standardized interfaces and repository occurred through no standardized messages with XML or JSON (JavaScript Object Notation) format over HTTPS (HyperText Transfer Protocol over Secure Socket Layer), while in the second case, it occurred through a direct database connection, invoking specific queries or stored procedures. In all cases, systems adopted property format to store data; therefore, the authors had to design and develop one RLUS web service and one IXS web server for each hospital or register/CDMS that ad hoc translated clinical and administrative contents from property format to standardized format and vice versa.

**Figure 1:**
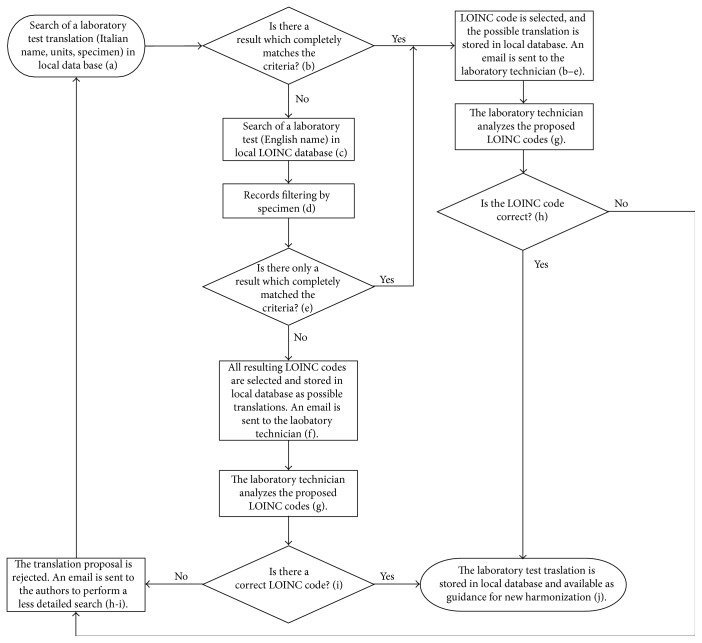
The harmonization algorithm.

This ad hoc standardization process started with a deep analysis of the structure and semantics of the information stored in the specific repository taken into account. The second step was to understand how to manage semantics. A terminological harmonization, with the support of laboratory technicians and supervisors of registers/CDMSs, between local terminology and LOINC was necessary. In this phase, the authors considered and modified an algorithm designed within a previous research collaboration [72]. The authors decided to adopt the translations between local terminologies and LOINC produced in the previous work as guidance for the new harmonization. In the same way, each new set of translations derived by the new harmonization would be guidance for the next and so on cyclically. The authors decided to design and develop a SQL Server database and a .NET web site to support the whole harmonization process. In detail, the database stored all the property of the concepts in the local terminologies (code, name, units, and specimen) and the corresponding LOINC code with the most relevant property (code, long common name, component, system, time aspect, property, scale type, method type, example ucum units, and example units), chosen during the harmonization loops. In addition, the database stored a copy of the LOINC database to perform local queries on LOINC. The authors extracted all distinct laboratory tests stored in the specific repository, and they analysed, with the physicians, which tests must be considered. For each of them, the resulting new harmonization algorithm was formed using the following steps (schematically reported in Figure 1):

- The authors searched through the web site for the Italian name and/or units and/or specimen of the test that must translate, and the tool returned the set of the concepts of other local terminologies, stored in the database, that were similar to the request and the corresponding LOINC code.
- If the authors found a test which completely matched the criteria they inserted it, the authors could select the LOINC code and the system stored the concept of the local terminology (with the property) and the selected LOINC code as a possible translation for the test (go to step “g”). The systems sent an email to the laboratory technician of the specific care facility or to the register/CDMS supervisor.
- If the system returned no result or no results was appropriate, the authors translated the Italian name of the test into English and searched for it in the local LOINC database copy through a specific form of the web site. Usually this research turned several records.
- The authors analysed these records, searching for the one or the ones that corresponded to the specimen of the test to be translated.
- If this process returned only one record, the authors selected this LOINC code as a possible translation for the test (go to step “g”). The system sent an email to the laboratory technician of the specific care facility or to the register/CDMS supervisor.
- If more than one record was found, the authors selected all the LOINC codes as possible translations that needed an expert's analysis. The system sent an email to the laboratory technician of the specific care facility or to the register/CDMS supervisor.
- The expert, that is, the laboratory technician or the register/CDMS supervisor, after receiving the email, could view the harmonization separately through a specific website page, proposed by the authors, that corresponded with an exact LOINC code (from steps “b” and “e”) or with more LOINC codes (from step “f”) (go to step “i”).
- In the event of the exact LOINC code, the expert could decide if the LOINC code was correct. If it was, they selected the LOINC code (go to step “j”); otherwise, they rejected the proposal. In this second case, the system sent an email to the authors (go to step “a” performing a less detailed search).
- In the case of multiple LOINC codes, the expert could select one LOINC code (go to step “j”) or no code. In this second case, the system sent an email to the authors (go to step “a” performing a less detailed search).
- The translation of the laboratory test, that is, code, name, units, and specimen of the local terminology and code, long common name, and the other relevant properties of LOINC, was available as guidance for the new harmonization of other repositories in research performed in step “a.”

The result of the harmonization process was a translation table.

The third step was the design of a specific algorithm to extract data from the repository and to create the CDA R2 containing the standardized laboratory report. The structure of each repository was different from the others, so the extraction mechanisms were specific for each solution, but the authors designed a generic algorithm that could be used to implement the responses of get() and list() functions of each RLUS web service, that is, the standardized laboratory reports:

- Identification of the required laboratory report
- Extraction of all administrative data
- Creation of the header of the CDA R2 document
- Extraction of all tests that formed the report
- Creation of the body of the CDA R2 document. For each test
  - if it corresponded to a single test, the authors created a section containing a single laboratory data processing entry;
  - if it was part of a battery, the authors searched for the other tests that formed the battery and then created a section containing a set of laboratory report item sections (including in turn one or more data processing entries).
- Translation of all codes defined in the local terminology in the corresponding LOINC code adopting the translation table previously mentioned. The LOINC code was inserted in the *translation* child element of *code* element.

The last step, which was performed only from the side of the registers and CDMS, was the design of a specific algorithm to extract the relevant information contained in a standardized laboratory report and to store the data in the specific repository. Also in this case as in the previous step, the fuelling mechanisms were specific for each solution, but the authors designed a generic algorithm that could be adopted to develop the put() capability of each RLUS web service of register/CDMS:

- Extraction of administrative data from the header of the CDA R2 document.
- Interaction with PIS to get the corresponding patient's identifier.
- Extraction of clinical data from the body of the CDA R2 document. For each section, the author extracted from it the test or the tests that it contains which must be considered.
- Translation of all LOINC codes reported in the *translation* child element of *code* element with the corresponding local code using the translation table previously mentioned.
- Storage in the repository.

Periodical focus groups were organized to discuss the state of the work and to collect feedback. The authors met two physicians, from each hospital involved every 3 months; medical doctors from other care facilities sometimes participated in these meetings as an audience, with an interest in becoming possible future partners in the project. They decided which processes to follow to obtain approval from the Ethical Committee; after development of the system, a test would be performed to validate the system, and, in the event of a positive feedback, the overall solution would be presented to the Ethical Committee.

## 4. Results

**Figure 2:**
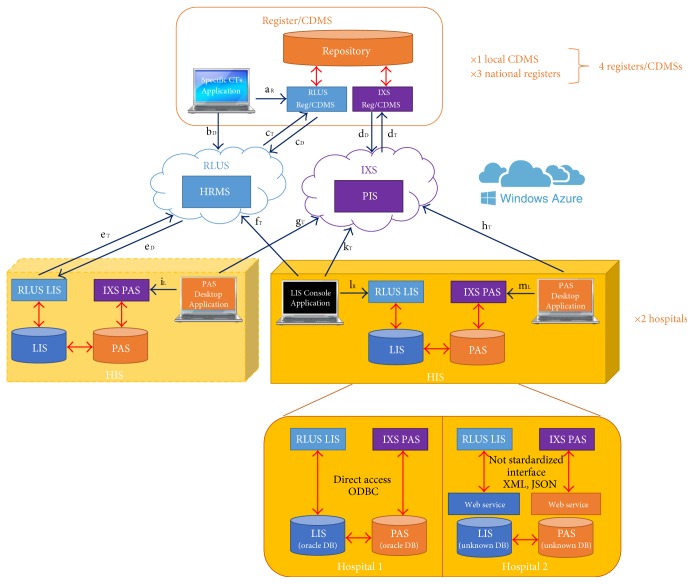
The designed and implemented architecture for the management of clinical trials on patients affected by infectious diseases. The light yellow box represents the solution to support the “Interoperable” Tier (adoptable after Ethical Committee approval), while the dark yellow box shows the system which is currently working (“Connected” Tier). Blue arrows represent the standardized call to web services, while red arrows represent not standardized communication adopting property formats. The additional information under the dark yellow box provides technical details about the solution that the authors designed and developed to connect the IXS and RLUS web services with the existing systems.

Figure 2 shows the architecture that the authors designed to achieve Tier 3, “Interoperable”, defined by the EHRCR Functional Profile Working Group. The solution was planned to support effective clinical data sharing between the actors involved in CTs which produce data, the HIS of care facilities, and those which process information, external clinical registers, or CDMSs of research centres. The core of this solution was formed by the HRMS and the PIS cloud node services (represented by 2 clouds) which automatically orchestrated the bidirectional communication between the HISs and CDMSs/registers. The HRMS, whose interface is compliant with the RLUS standard, is responsible for managing clinical data; the PIS, whose interface is compliant with the IXS standard, has the same purpose for administrative data.

As previously mentioned, the components of each HIS considered were the LIS and the PAS, as represented in the light yellow box in Figure 2, which store clinical and administrative data, respectively. From the research centre side, a CDMS or register was formed by a repository, which stores all information collected in previous CTs.

In order to connect the node services with the HIS and the register/CDMS, all systems were located in the same network (internet) allowing patient's data sharing among these actors. In addition, the authors planned the implementation of two types of agents: web services and client applications. The web services were adopted as passive actors to provide access to the content, on the one side, of the registry and CDMS repositories and, on the other side, of the EHR and PAS. The same HSSP standards were adopted for the interface designs of the web services: IXS to manage administrative data and RLUS to exchange clinical information (purple “IXS” and blue “RLUS” rectangles in Figure 2). The other class of software agents, client applications (represented as laptop computers in Figure 2), was included as architecture active components to trigger two categories of events: the management of administrative information and the study of clinical information to get the results of CTs. In the first case, the “PAS Desktop Application,” which worked on behalf of the hospital, was responsible for the management of patient enrolment/disenrolment from CTs and patient transfer from one hospital to another. In the second case, the “Specific CTs Application,” working on behalf of the research centres, was used to perform the particular analysis of CTs on clinical data, stored both within the repository and LIS, in run-time. It is important to highlight that the adoption of standardized interfaces allows these client applications to interact with both the node cloud services and the web services (blue arrows between PAS Desktop Application and IXS PAS, between PAS Desktop Application and PIS, between Specific CTs Application and RLUS Reg/CDMS, and between Specific CTs Application and HMRS). In fact, each client adopts the same interfaces to communicate with node cloud services and the web services because it is compliant to IXS, in the case of the PAS Desktop Application, and RLUS, in the case of the Specific CTs Application. The only element that the client must change during the call of a capability is the endpoint of the service.

**Figure 3:**
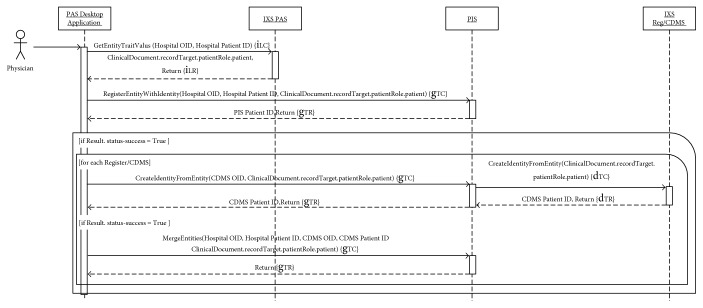
Sequence diagram of a patient's enrolment. The content inside the curly brackets refers to the blue arrows reported in Figure 2. The second subscript letters distinguish the call (C) of specific web service functions to the response (R).

**Figure 4:**
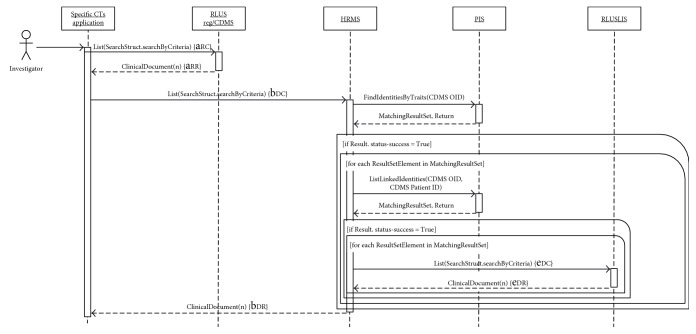
Sequence diagram of run-time analysis of clinical information. The content inside the curly brackets refers to the blue arrows reported in Figure 2. The second subscript letters distinguish the call (C) of specific web service functions to the response (R).

In order to allow the reader to completely understand the complex workflow orchestrated by the HRMS and PIS, working as intermediaries for other RLUS and IXS web services, the authors propose two storyboards which explain typical scenarios: the patient's enrolment (Figure 3) and the run-time analysis of clinical information (Figure 4).

When a patient is considered suitable for research purposes, the physician can select the specific CDMSs and registers in which they want to enrol the patient, using the PAS Desktop Application. First, the application interacts with the IXS interface of PAS (IXS PAS) to obtain the administrative data, encapsulated within a Patient class instance of the CDA R2 document, by calling the GetEntityTraitValues() capabilities. It then deidentifies the received HL7 v3 message in order to anonymize the information, only maintaining the birth year, gender, nationality, and race. After that, it calls the RegisterEntityWithIdentity() capability of PIS to sign up the patient within the PIS, sending the care facility Object Identifier (OID), the patient ID (identifier), and the deidentified information. The PIS processes this request, and if it is correct, it stores the administrative data and returns the identifier, which is associated to the specific patient, with information on the state of the operation. If successful, the PAS Desktop Application, for each register or CDMS selected by the physician, calls the CreateIdentityFromEntity() operation of the PIS indicating the OID of the research study. When the PIS receives this information, as mediator, it in turn sends the same request to the IXS interface of the specific register or CDMS (IXS Reg/CDMS). In this way, a new entity with the same administrative patient's data is created within the repository of the specific register/CDMS and the corresponding identifier is returned first to the PIS and then to the application. In this way, the PAS Desktop Application created a new identifier for each register/CDMS. The last step links all these identifiers assigned to the patient within the PIS in the different systems involved. This can be performed by calling the MergeEntities() functionality of PIS specifying the patient ID within the PAS and the register/CDMS, with the corresponding OID of the source which generated them. Thanks to merge/unmerge capabilities provided by the IXS standard, the PIS is able to manage different types of situations, using similar mechanisms, such as the patient's transfer from one hospital to another or the patient's disenrolment from the CT.

**Figure 5:**
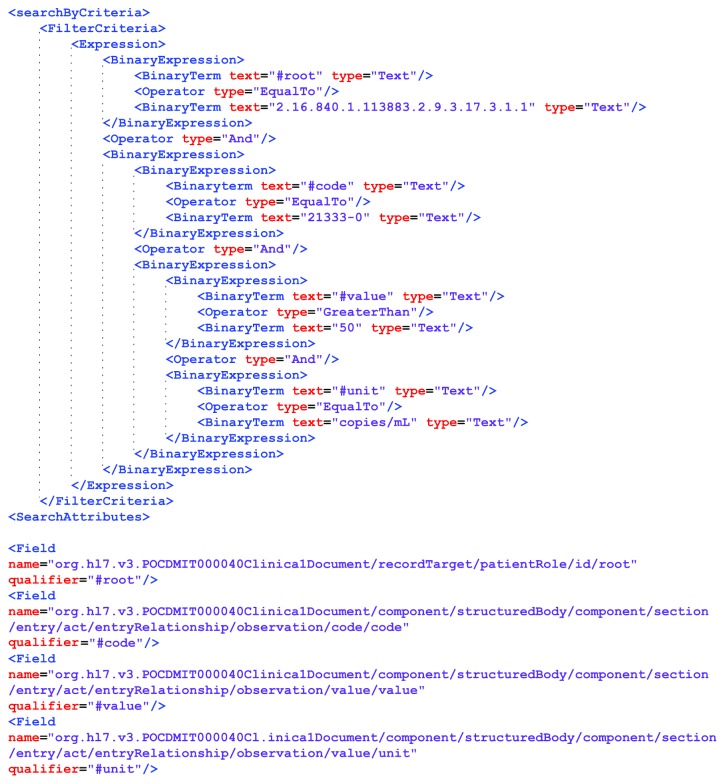
Sample extract of a SOAP request which contains the RLUS object to map the filter criteria.

Figure 4 represents the sequence diagram of the interactions which occur when an investigator wants to perform a run-time analysis on clinical and administrative data. Using the Specific CTs Application, the investigator can select the criteria needed for the required study. The client application calls List() capability of the RLUS interface of the repository (RLUS Reg/CDMS) and then of the HRMS. This step allows the selection all CDA R2 documents that match the specific query among all documents stored both in the repository of the register/CDMS and in the LIS of the involved hospitals. An extract of a SOAP request that contains the RLUS object to map the filter criteria adopted as input parameter of List() function is shown in Figure 5. In detail, it represents a query to obtain clinical data of patients whose pharmacological suppression is not effective. The FilterCriteria element contains an expression that an RLUS interface can interpret to indicate that the clinical documents requested must be related to patients enrolled in the specific CT (indicated by the root attribute of the ID element of the patientRole element of the clinical document which must correspond to the OID of the CT) and must contain observations of concentration of HIV RNA (RiboNucleic Acid) (LOINC code 21333-0) greater than 50 copies/mL. When the client calls the List() capability of the RLUS Reg/CDMS, the service returns a list of all clinical documents that are stored in the repository and match the indicated criteria. When the application asks the HRMS for the same operation, first, this node service interacts with the PIS to obtain the list of all patients enrolled in the specific CT indicated, as mentioned above, by the specific OID, by calling the FindIdentitiesByTraits() capability of PIS. If this operation was successful, for each patient returned, the HRMS asks the PIS for a list of all identities (Hospital OID, Patient ID) linked the indicated patients. Also, if this list is full, for each identity, the HRMS interacts with the RLUS interface of the LIS (RLUS LIS) of the specific care facility (indicated by the Hospital OID) to get all deidentified clinical documents related to the specific patient (indicated by the Patient ID) which match the criteria indicated by the investigator. In this case, the HRMS changes the FilterCriteria replacing, in the first BinaryExpression, the filter on the root attribute of the ID element of patientRole with the extension attribute of the same element, to indicate the Patient ID. After, the Specific CTs Application has retrieved all the CDA R2 documents, and it is able to perform the required analysis without any data duplication.

At the beginning of 2013, the authors started to implement a solution based on this architecture to support the management of clinical trials on patients affected by infectious diseases. The authors began the development of the HRMS and PIS core services, which are represented by two WCF services, to be hosted on the private cloud Microsoft Azure. For the management of administrative data, the authors developed the database, mentioned previously, hosted on Microsoft SQL Azure connected with the PIS. Then, they focused on the design and implementation of the RLUS and IXS interfaces of four register/CDMS (a local CDMS, three national registries) and two HIS of two different care facilities, all represented by WCF services that connect to objects made available by the hospital teams. These agents are responsible for the bidirectional ad hoc translation of clinical and administrative content from the standardized format (CDA R2) to proprietary format (specific for each repository, LIS and PAS) and vice versa.

**Figure 6:**
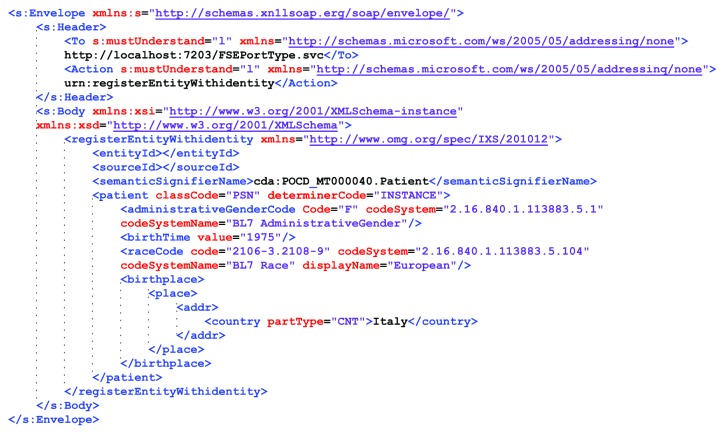
Example of a SOAP message traced during the second interaction of the diagram represented in Figure 3 between the PAS Desktop Application of a hospital involved and the PIS node service.

The last step was the implementation of the active agents, the CTs Specific Applications, and the PAS Desktop Application, which were connected with the implemented standardized interfaces and the node services.  Figure 6 shows an example of a SOAP message traced during the second interaction of the diagram represented in Figure 3 between the PAS Desktop Application of a hospital involved and the PIS node service. This XML file explains how the solution effectively supports interoperability at all levels. The technical interoperability is guaranteed by the SOAP message structure in which the header indicates the receiver (the PIS node service) and the action that the application requested to the service. The semantic interoperability is provided by the adoption of a standardized semantic signifier (POCD_MT000040.Patient object of CDA R2), which in the reported example indicates a 40-year-old, European female patient, born in Italy. Finally, process interoperability is guaranteed by the standardized name of the SOAP action (the string “urn:registerEntityWithIdentity”) and structure of body content (the xml element registerEntityWithIdentity), which indicates the specific Identity (an Entity ID - Source ID pair) that must be registered with the specific traits indicated in the semantic signifier.

Figure 2 shows the overall architecture that the authors implemented to manage CTs on patients affected by infectious diseases. From the side of the hospitals, the light yellow box represents the solution that was developed to connect the HIS with the overall architecture in order to support the “Interoperable” Tier. To preliminary test if the HRMS and PIS were able to orchestrate the communication among all actors, a simulated scenario was set up at the end of 2013. An instance of HRMS and PIS was hosted on a specific subscription of Microsoft Azure. In order to test this system, the HIS administrators of the two hospitals made available an instance of LIS and PAS, installed in parallel to the real systems. Ten simulated nonhospitalized patients from each hospital (for a total of 20) were enrolled in a CT. For each patient, fake clinical data were manually inserted into the LIS instances in order to allow it to perform research on information from 2012. In detail, the authors considered the mean values observed in a clinical practice of involved care facilities: a nonhospitalized HIV patient performs on average a complete laboratory test each 3 months, and a complete laboratory report contains about 50 observed clinical parameters. Therefore, 160 simulated laboratory reports, for a total 8000 values, were made available. This solution was tested for 2 months by 4 physicians, who expressed great satisfaction during a periodical focus group.

This very preliminary study was presented to the Ethical Committee, the administrators of the HIS involved care facilities, and hospital policy makers, to give consent to external systems, hosted outside the HIS, to directly access the LIS and PAS contents, through the implemented standardized interfaces, in order to perform more relevant tests. Due to great administrative delay, which will be discussed in the next section, the physicians involved, enthusiastic about the tested solution, asked authors if it was possible to set up a temporary system in order to allow them to participate in CTs awaiting approval, during one of the periodical focus groups. For this reason, the authors decided to temporarily simplify the architecture on the side of the HIS to only support the “Connected” level indicated by the EHRCR Functional Profile Working Group (see dark yellow box in Figure 2). The idea was to provisionally add an active client agent within the HIS, the “LIS Console Application,” which extracts new clinical data daily related to patients enrolled in CTs and updates the repository of the corresponding registers/CDMSs. In detail, this type of client was designed to be installed within the hospital LAN (Local Area Network) in order to extract the new clinical content stored in the LIS daily, through the implemented RLUS interface, and send it to the repository of the selected registries/CDMSs, through the HRMS. In this way, the direct access of LIS clinical content was still denied to systems hosted outside the HIS and therefore the solution could be more quickly approved by the Ethical Committee, the HIS administrators, and hospital policy makers.

**Figure 7:**
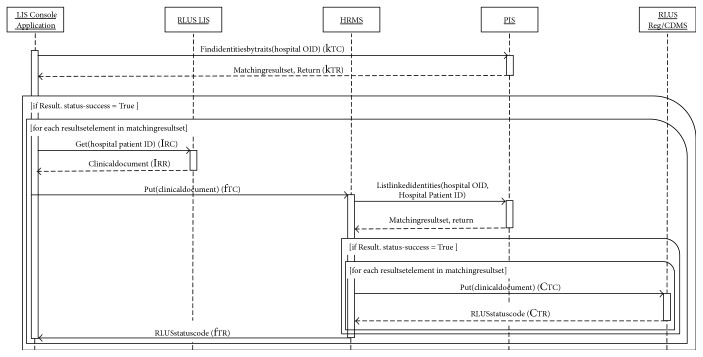
Sequence diagram of the daily automatic update of repository of CDMSs/registers. The content inside the curly brackets refers to the blue arrows reported in Figure 2. The second subscript letters distinguish the call (C) of specific web service functions to the response (R).

Figure 7 explains how the described architecture can also manage this daily workflow. Each night, the LIS Console Application calls the PIS FindIdentitiesByTraits() capability to obtain an ID list, for all patients participating in one or more research studies involved in the architecture, which is stored within the specific HIS. For each ID, the application interacts with the RLUS LIS, to obtain, if present, the new laboratory report of the corresponding patient, by calling the Get() capability, and sends it to the HRMS using the Put() operation. From this CDA R2 document, the HRMS extracts the patient's identifier (hospital OID and patient ID) and asks the PIS for all other patient ID, together with the corresponding source, which are linked with the extracted patient identifier (ListLinkedIdentities()). For each result, the HRMS replaces ClinicalDocument.recordTarget.patientRole.patient.id with the information supplied by the PIS and sends the report to the RLUS Reg/CDMS, which is responsible for the storage of clinical data in the specific repository.

This “Connected” scenario was submitted to the Committee which approved its implementation in both hospitals. This authority requested a testing phase of 3 months on real consenting patients before applying it in clinical practice.

**Figure 8:**
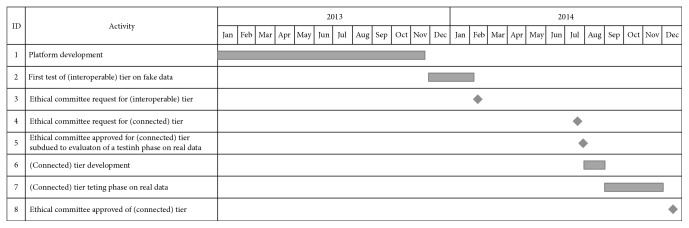
The temporal evolution of the project.

Afterwards, the LIS Console Application was developed and introduced in the solution which was previously set up; in addition, the communication between each Specific CTs Application was temporarily remove because all data were already available in the repository of the specific register/CDMS, after the daily update mentioned above. In order to test this system, 65 hospitalized and nonhospitalized patients for each care facility (for a total of 130) were considered. A hospitalized HIV patient performs blood test every day, and the resulting laboratory report contains about 20 clinical observations. At the end of this period, 5590 values were exchanged. The mean rate of exam/time[minutes] was calculated for both hospitals; in the case of RLUS repository access through a nonstandardized interface, this value was 0.79 while in case of RLUS direct access to the LIS database it was 1.26. The authors integrated two forms to the PAS Desktop Application with two different aims. The first one was implemented to allow physicians to perform a retrospective and manual comparison between the clinical content of the LIS related to the simulated patients, and the information automatically extracted and stored in the repository of the involved registers/CDMSs. A random sample of 5% of all values exchanged in the test period was validated, and no errors were detected. The second form was designed to collect feedback from all medical staff: it allowed physician to choose their satisfaction level (quantifiable from 1 to 10) and to optionally indicate more detailed information. The mean satisfaction level measured was 9.6, and the reason was that the system reduced the workload for the management of clinical trials. The results of the manual comparison and the physician feedback were submitted to the Ethical Committee, the HIS administrators, and hospital policy makers in order to adopt the “Connected” configuration of the architecture during the daily clinical routine. The authors asked these authorities to allow LIS Console Application to have reading permission towards the LIS content; both systems, LIS Console Application and LIS, are hosted inside the HIS and communicate through a standard a RLUS LIS interface. The anonymous clinical data transfer from the PAS to the repository of the specific register/CDMS, which is orchestrated by the HRMS core service hosted in a private Microsoft Azure cloud, also has to be approved. Administrative data sharing is performed in the same way for both the “Connected” and “Interoperable” Tiers, involving the PAS Desktop Application and the PIS core service as previously described. The authorization to support administrative data exchange from the LIS to the repository of the specific register/CDMS, orchestrated by the PIS core service hosted in Microsoft Azure, has been requested too. In the “Connected” scenario, the LIS Console Application requires reading access to LIS only to systems within the HIS. For this reason, the Ethical Committee request was quickly approved, and the solution presented here is currently in use.  Figure 8 presents the temporal evolution of the project in a schematic view.

At present, the clinical and administrative data of 2610 patients are managed by this solution; in detail, 474 clinical parameters and 2,441,717 values have been exchanged. The system was set up at the end of 2014, but thanks to a mechanism similar to the one proposed in Figure 7, it allowed the recovery of all information stored in the PAS and LIS since 2008.

## 5. Discussion

The whole solution was structured on the requirements suggested by the eSDI group [7] and on health informatics standards to completely support interoperability. Thanks to the adoption of common standardized interfaces, provided by all repositories involved and the core node services, the architecture is able to share all information with other external systems. For example, an external Decision Support System can have access to specific clinical content managed within the architecture, by calling the Get() or List() capability, provided by the HRMS node service, indicating the filter criteria that matches the particular needs. The strict adherence to international standards required a great implementation effort in the development of the first application in each single hospital and register/CDMS. In fact, both care facilities and research centres adopted nonstandardized solutions that obligated the authors to design and implement ad hoc standardized interfaces to translate clinical and administrative content from the standardized format to proprietary format. Furthermore, a continuous deep collaboration with the HIS and register/CDMS repository administrators, which caused an increase in their workload, was fundamental and valuable to realize this system. Still, this initial investment guarantees a more rapid extensibility of the solution, which at present is limited to a single ward of care facilities, to include more wards until the whole hospital is covered. This will be possible since the designed application is directly connected with the LIS and PAS belonging to the HIS.

Full adoption of the standards will allow extension of the connection outwards from the care institutions involved towards other research networks which investigate different diseases. Unfortunately, the application of this architecture to support CTs in other pathologies depends on the availability of CDA R2 Implementation Guides; in fact, as mentioned previously, this architecture could not be implemented in the research of degenerative eye diseases due to the absence of IGs for the specific medical visits. The HL7 and its affiliates are helping to create working groups aimed at analysing the necessary specific context and at preparing the draft for new white papers. In addition, while HSSP standards are distributed through both human readable specification documents and computer processable files, CDA R2 IGs are only formed by white papers. This requires human interpretation of the specifications that can be difficult and create errors. The adoption of a tool suite that supports the creation and maintenance of HL7 templates, value sets, scenarios, and data sets as ART-DÉCOR [73] could be useful for the design and implementation of solutions able to solve these issues (e.g., CDA R2 validation servers).

The architecture proposed in this paper can support the adoption of all other XML-based standards that support semantic interoperability in health informatics. This is possible, thanks to the dynamic mechanism of semantic signifier management provided by HSSP products. In particular, the authors are planning to adopt the emerging HL7 messaging Version FHIR (Fast Healthcare Interoperability Resources), which was specifically promoted to support data sharing.

As previously mentioned, even if the described architecture only allows realisation of the “Interoperable” Tier, the solution that is currently running, for the present hospital policies, is limited to the “Connected” level, which extracts the clinical content stored in LIS daily, and sends it to the repository of the selected registries/CDMSs.

In this “Connected” implementation, which involves two HIS and four registries/CDMSs, the instance of the Microsoft Azure cloud is able to manage the workflow daily requiring an extraction time much shorter than one night. This amount of time is compatible with the medical practice; therefore, load balancing mechanisms, provided by the Azure platform, to enable scalability, were not yet adopted. The head physicians of 10 care facilities in another Italian region were asked to participate in this solution too. In order to be able to involve these other HISs, load balancing will be considerate and specific performance tests will be carried out. This phase will be necessary to research in literature studies which will be the base to choose the cloud configurations to improve performance limiting costs. In fact, Tudoran et al. stated “that the right cloud configuration can improve overall application performance by as much as three times and can significantly reduce the cost” [74]. Another aspect which will be considered is that the extraction time of a standardized laboratory report also depends on the technical features of the other involved systems on the side of both HIS and registries/CDMSs. The meant rates of exam/time [time] calculated showed that the solution which adopted a RLUS LIS interface directly connected with LIS is significantly faster (59.5%) than the one using nonstandardized interface. For these reasons, the authors will propose to adopt solutions adopting the direct connection approach. Finally, in order to support either the “Connected” or “Interoperable” level would be necessary to improve the capability of the repository of the involved LISs or registries/CDMSs.

In the working group responsible for the definition of functional requirements for the Italian PHR, there were a lot of discussions about data sharing convenience temporarily falling back on data reuse. This represents an opening with respect to all the absolute oppositions imposed by the patient's right to data privacy, delegated to all hypotheses of data reuse adopted for the first prototype of regional PHR, previously implemented. This opening is caused by the good intuition of Italian legislation that explicitly indicates that one of the aims of the establishment of PHR was for medical, biomedical, and epidemiologic studies and research, as previously mentioned [35]. Moreover, in September 2015, a new act from the Italian Prime Minister stated, in a clearer way, the possibility of using clinically collected data for research purposes (providing their correct anonymization) even with the use of external information technology services [36]. This would help in convincing the hospital policy makers to accept the full use of the proposed platform.

After the described testing phases, the medical staff of both hospitals expressed great satisfaction regarding the implemented system, as attested by the feedback collected through the second form mentioned in the Results (mean satisfaction level 9.6/10), since it reduced the workload for the management of clinical trials. After the testing phase of the “Connected” Tier solution, on a random sample of 5% of inserted clinical values related to patients of both hospitals, a retrospective and manual comparison between the HIS and database was carried out, using the specific form of the PAS Desktop Application, with no errors detected. After this comparison, the results were submitted to the local Ethics Committee, and the solution was definitively approved for being used in clinical practice.

## Figures and Tables

**Table 1:** Description of the RLUS standard functionalities provided by the RLUS Management and Query Interface.

Capability	Aim	Input parameters	Output parameters	Summarized description
Get ()	To retrieve a single health information resource, mapped through a specific semantic signifier, which fulfills the specific query performed by the client.	An instance of RLUSSearchStruct (a well-defined and machine readable data structure to describe a search based on filter criteria or examples and the name of the semantic signifier to retrieve). An instance of RLUSLogicalRecordID (a structure that identifies a logical record managed by RLUS).	An instance of RLUSStatusCode (a structure to contain the code indicating whether the operation was successful or not and a corresponding message). The RLUSResultSet (the requested resource only if it exists and if it is unique).	It is called by clients to get a resource with specific features. The client indicates either (1) the search criteria or (2) the resource identifier. The service (1) indicates if any errors occurred and (2) returns the required resource if it exists and if it is unique.
List()	To retrieve a list of health information resources, mapped through a specific semantic signifier, which fulfills the specific query performed by the client.	An instance of RLUSSearchStruct. The maxResultStreams (the maximum number of set of resources to be returned, that is, the maximum number of call that must be performed to get all resources). The previousResultID (the ID token returned by a previous call).	An instance of RLUSStatusCode. The RLUSResultSet (an array formed by the requested resources). The resultID (the ID token that identifies a streaming result set). The finishedFlag (a flag that indicates if all resources were retrieved or the approximate number of call that must be performed to get all resource).	It is called by clients to get a list of resources with specific features. The client indicates (1) the search criteria, (2) the maximum number of call that must be performed to get all resources, and (3) the ID token returned by a previous List () call, if it was called. The service (1) reports if any errors occurred, (2) returns the list of requested resources if exist, (3) the ID token, and (4) indicates if all resources were retrieved or how many calls must be performed to get all resources.
Locate()	To retrieve a list of RLUS service locations where the desired health information resources can be found.	An instance of RLUSSearchStruct. The maxResultStreams. The previousResultID.	An instance of RLUSStatusCode. The RLUSLocationsResultSet (an array formed by the RLUS service locations where the resources can be found). The resultID. The finishedFlag.	It is called by clients to get a list of RLUS service locations where the resources with specific features can be found. The client indicates (1) the search criteria, (2) the maximum number of call that must be performed to get all locations, and (3) the ID token returned by a previous Locate() call, if it was called. The service (1) reports if any errors occurred, (2) returns the list of requested locations if exist, (3) the ID token, and (4) indicates if all locations were retrieved or how many calls must be performed to get all locations.
Put()	To store an instance of a logical record in the repository.	An instance of RLUSWriteCommandEnum (a structure to contain an enumeration indicating the action the service must perform: insert only, update only, or “upsert.” “Upsert” means that first the service has to check whether the resource already exists, and then, if so, it executes an update, but if not, it executes an insert). An instance of RLUSPutRequestSrcStruct (a structure to contain the semantic signifier, which maps the logical record, security, source, and network address context of the caller of the Discard() operation which is necessary to retrieve data to clean the RLUS and audit logs). The RLUSResultSet.	An instance of RLUSStatusCode. An instance of RLUSLogicalReco-rdID.	It is called by clients to store a resource within the repository of the service. The client indicates (1) the right action that the service must perform, (2) some security, source, and network address information, and (3) the resource that must be stored. The service (1) indicates if any errors occurred and (2) returns the resource identifier.
Discard()	To either physically or logically delete resources from the repository.	An instance of RLUSSearchStruct. An instance of RLUSUpdateRequestSrcSt-ruct (a structure logically equivalent to RLUSPutRequestSrcStruct).	An instance of RLUSStatusCode.	It is called by clients to physically or logically delete resources with specific features from the repository of the service. The client indicates (1) the search criteria and (2) some security, source, and network address information. The service (1) indicates if any errors occurred.
Describe()	To get the detailed schema definition of the required semantic signifier.	The semanticSignifierName (a human readable text string that corresponds to the name of the semantic signifier).	An instance of RLUSStatusCode. An instance of RLUSSemanticSi-gnifier (a meta-data structure to define the semantic signifier data type processed by RLUS).	It is called by clients to get the detailed schema definition of a specific semantic signifier. The client indicates (1) the semantic signifier name. The service (1) indicates if any errors occurred and (2) provides the corresponding detailed schema definition.

**Table 2:** Description of the IXS standard functionalities provided by the IXS Management and Query Interface and the IXS Admin Editor Interface.

Capability		Aim	Input parameters	Output parameters	Summarized description
IXSManagementAndQueryInterface	RegisterEntityWithIdentity()	To register a new entity (the software representation of a real word entity), with specific traits which describe it, associated with a specific identity (an Entity ID - Source ID pair).	A pair of entity ID (an identifier associated with an entity) and the specific source ID (an identifier associated with the source, that is, the system which generates the entity). An instance of the IXS Semantic Signifier which provides all the traits. The name of the semantic signifier adopted.	The IXS ID (an identifier within the IXS) which is associated with the registered identity. An instance of the IXS Status (a structure to contain a flag indicating whether the operation was successful or not and a structure representing all the corresponding conditions which occurred during the operation call).	It is called by clients to register a new entity with specific features within the service. The client indicates (1) the external entity identifier, (2) the system in which the identifier is defined, and (3) the features mapped within an instance of (4) the indicated semantic signifier. The service returns (1) an internal identifier associated to the entity and (2) indicates if any errors occurred.
	CreateIdentityFromEntity()	To register a new identity, with specific traits, on behalf of a source which cannot generate entity IDs.	The specific Source ID of the identity to create. An instance of the IXS Semantic Signifier which describes all the traits. The name of the semantic signifier adopted.	The entity ID generated and associated with the entity. An instance of the IXS Status.	It is called by clients to generate an identifier for an entity on behalf of a system that cannot create identifiers. The client indicates (1) the system in which it wants to create the identifier and (2) the features of the entity mapped within an instance of (3) the indicated semantic signifier. The service returns (1) the identifier generated for the system indicated by the client and (2) reports if any errors occurred.
	UpdateEntityTraitValues()	To update the traits associated with a specific identity.	The entity ID. The source ID. An instance of the IXS Update Qualifier (a structure to contain qualifier to limit, constrain, or otherwise alter the behaviour of the update). An instance of the IXS Semantic Signifier which provides all the traits to update. The name of the semantic signifier adopted.	An instance of the IXS Status.	It is called by clients to update the features of a specific entity within the service. The client indicates (1) the entity identifier, (2) the system in which the identifier is defined, (3) the features to update, mapped within an instance of (4) the indicated semantic signifier, and (5) other information about the way in which the service have to update the features (e.g., if to replace the entire set of features with the one indicated by the client or to update only the indicated features). In return, the service (1) indicates if any errors occurred.
	RemoveIdentity()	To remove the identity with its associated traits from the service.	The entity ID. The source ID. An instance of the IXS Semantic Signifier which provides all the traits of the identity. The name of the semantic signifier adopted.	An instance of the IXS Status.	It is called by clients to remove a specific entity from the service. The client indicates (1) the entity identifier, (2) the system in which the identifier is defined, and (3) the associated features, mapped within an instance of (4) the indicated semantic signifier. In return, the service (1) indicates if any errors occurred.
	GetEntityTraitValues()	To retrieve all traits associated with a specific identity.	The entity ID. The source ID. The name of the semantic signifier adopted.	An instance of the IXS Semantic Signifier which provides all the traits. An instance of IXS Status.	It is called by clients to get all features associated with a specific entity. The client indicates (1) the entity identifier, (2) the system in which the identifier is defined, and (3) the semantic signifier which it wants to be adopted by the service to map the required features. The service returns (1) an instance of the indicated semantic signifier that contains all features and (2) indicates if any errors occurred.
	FindIdentitiesByTraits()	To retrieve all identities within the IXS which match some specified criteria.	The entity ID, if known. The source ID, if known. The IXS ID, if known. An optional instance of the IXS Search Qualifier (a structure to contain qualifier to perform more detailed search). An instance of the IXS Semantic Signifier which provides all the traits. The name of the semantic signifier adopted.	An instance of the IXS Result Set (a structure to contain the list of search results). An instance of the IXS Status.	It is called by clients to retrieve all identifiers of entities whose features match some criteria. The client indicates the criteria which can be (1) an entity identifier, (2) a system in which the identifiers must be defined, (3) the entity identifier defined within the service, (4) other search qualifiers (e.g., the match algorithm to perform the query, the max number of search results), and (5) the features mapped within an instance of (6) the indicated semantic signifier. The service returns (1) a list of search results which match the indicated criteria and (2) indicates if any errors occurred.
	ListLinkedIdentities()	To retrieve all identities which are linked to the provided identity.	The entity ID. The source ID. An optional instance of the IXS Search Qualifier. An instance of the IXS Semantic Signifier which provides all the traits. The name of the semantic signifier adopted.	An instance of the IXS Result Set. An instance of the IXS Status.	It is called by clients to retrieve all identifiers that are linked with the entity/entities whose features match some criteria. The client indicates (1) the entity identifier, (2) the system in which the identifier is defined, (3) other search qualifier, and (4) the features mapped within an instance of (5) the indicated semantic signifier. The service returns (1) a list of search results which match the indicated criteria and (2) indicates if any errors occurred.
	ListUnlinkedIdentities()	To retrieve all identities which are not linked to the provided identity, but which have the same features.	The entity ID. The source ID. An optional instance of the IXS Search Qualifier. An instance of the IXS Semantic Signifier which provides all the traits. The name of the semantic signifier adopted.	An instance of the Result Set. An instance of the IXS Status.	It is called by clients to retrieve all identifiers which identify the entity/entities with specific features, but that are not linked with the provided identifier. The client indicates (1) the entity identifier, (2) the system in which the identifier is defined, (3) other search qualifier, and (4) the features mapped within an instance of (5) the indicated semantic signifier. The service returns (1) a list of search results and (2) indicates if any errors occurred.
IXSAdminEditorInterface	LinkEntities()	To link a source identity with a target one.	The source Identity Entity ID. The source Identity Source ID. The target Identity Entity ID. The target Identity Source ID. The code of the reason for making the link. An instance of the IXS Semantic Signifier which provides all the traits. The name of the semantic signifier adopted.	An instance of IXS Status.	It is called by clients to create an explicit linking between identifiers which represent entities with the same specific features. The client indicates (1) the identifier of the source entity, (2) the system in which the identifier of the source entity is defined, (3) the identifier of the target entity, (4) the system in which the identifier of the target entity is defined, (5) the code which corresponds to the reason of the link, and (6) the features mapped within an instance of (7) the indicated semantic signifier. In return, the service (1) indicates if any errors occurred.
	UnlinkEntities()	To remove the link between a source identity and a target one.	The source Identity Entity ID. The source Identity Source ID. The target Identity Entity ID. The target Identity Source ID. The code of the reason for removing the link. An instance of the IXS Semantic Signifier which provides all the traits. The name of the semantic signifier adopted.	An instance of the IXS Status.	It is called by clients to break an explicit linking between identifiers which represent entities with the same specific features. The client indicates (1) the identifier of the source entity, (2) the system in which the identifier of the source entity is defined, (3) the identifier of the target entity, (4) the system in which the identifier of the target entity is defined, (5) the code which corresponds to the reason of the breaking and (6) the features mapped within an instance of (7) the indicated semantic signifier. In return, the service (1) indicates if any errors occurred.
	MergeEntities()	To merge a source identity into a target one.	The source Identity Entity ID. The source Identity Source ID. The target Identity Entity ID. The target Identity Source ID. The code of the reason for making the merge. An instance of IXS Semantic Signifier which provides all the traits. The name of the semantic signifier adopted.	An instance of the IXS Status.	It is called by clients to completely merge identifiers which represent entities with the same specific features. The client indicates (1) the identifier of the source entity, (2) the system in which the identifier of the source entity is defined, (3) the identifier of the target entity, (4) the system in which the identifier of the target entity is defined, (5) the code which corresponds to the reason of the merge, and (6) the features mapped within an instance of (7) the indicated semantic signifier. In return, the service (1) indicates if any errors occurred.
	UnMergeEntities()	To separate a source identity from a target one.	The source Identity Entity ID. The source Identity Source ID. The target Identity Entity ID. The target Identity Source ID. The code of the reason for the separation of the identities. An instance of the IXS Semantic Signifier which provides all the traits. The name of the semantic signifier adopted.	An instance of the IXS Status.	It is called by clients to separate identifiers which represent entities with the same specific features. The client indicates (1) the identifier of the source entity, (2) the system in which the identifier of the source entity is defined, (3) the identifier of the target entity, (4) the system in which the identifier of the target entity is defined, (5) the code which corresponds to the reason of the separation, and (6) the features mapped within an instance of (7) the indicated semantic signifier. In return, the service (1) indicates if any errors occurred.
	ActivateEntity()	To activate an identity.	The entity ID. The source ID. The code of the reason for the activation of the identity. An instance of the IXS Semantic Signifier which provides all the traits of the identity. The name of the semantic signifier adopted.	An instance of the IXS Status.	It is called by clients to activate an entity with specific features. The client indicates (1) the entity identifier, (2) the system in which the entity identifier is defined, (3) the code which corresponds to the reason of the activation, and (4) the features mapped within an instance of (5) the indicated semantic signifier. In return, the service (1) indicates if any errors occurred.
	DeactivateEntity()	To deactivate an identity.	The entity ID. The source ID. The code of the reason for the inactivation of the identity. An instance of the IXS Semantic Signifier which provides all the traits of the identity. The name of the semantic signifier adopted.	An instance of the IXS Status.	It is called by clients to deactivate an entity with specific features. The client indicates (1) the entity identifier, (2) the system in which the entity identifier is defined, (3) the code which corresponds to the reason of the inactivation, and (4) the features mapped within an instance of (5) the indicated semantic signifier. In return, the service (1) indicates if any errors occurred.
